# First three cases of scalp temperature change in symptomatic areas affected by nummular headache: a case report

**DOI:** 10.1186/s12883-018-1231-0

**Published:** 2018-12-28

**Authors:** Yonghui Liu, Tianlu Wei

**Affiliations:** Department of Encephalopathy, The First Affiliated Hospital of Guangxi University of Chinese Medicine, Nanning, 530023 China

**Keywords:** Nummular headache, Coin-shaped cephalalgia, Paraesthesia

## Abstract

**Background:**

Nummular headache is a distinct headache disorder characterized by a rounded or elliptical symptomatic area that is typically 2 to 6 cm in diameter and does not change in shape or size with time. Although the pathomechanism is still not clear, nummular headache is thought to be a primary headache disorder. To date, more than 250 cases have been reported; the symptoms of this disease vary, but no cases with scalp temperature changes in the symptomatic areas have been reported yet. In this study, we present three patients with a new manifestation of nummular headache, in which the symptomatic areas of the scalp were colder or warmer than normal areas; we believe that our work might be helpful for medical practitioners and researchers.

**Case presentation:**

The temperature differences between the symptomatic areas and the normal areas were tested in three patients with nummular headache accompanied by changes in scalp temperature. Three patients’ symptomatic areas were either colder or warmer than the normal areas. In every case, we took measurements from the painful site and from the opposite side of the head. The margin of error was 0.01 °C, and the difference was statistically significant (*P* < 0.01).

**Conclusion:**

We firmly believe that our study will provide an enriched understanding of the variation in clinical manifestations of nummular headache. Our observations might also have clinical implications regarding the pathomechanism of this disease, which remains largely unclear at present.

## Background

Nummular headache, or coin-shaped cephalalgia, is a headache disorder characterized by pain in a round or oval/elliptical shaped area of the head; this disorder was first described in 2002 by Pareja in a series of 13 patients, in whom the symptomatic area did not change in shape or size over time [1]. Since 2002, more than 250 cases have been reported in the literature [2]. With the increasing attention being paid to nummular headache, new clinical manifestations have been reported, such as bifocal [3–5] and even multifocal localization [6, 7], trophic change [7, 8], menstruation-related timing [9], and occurrence secondary to subtentorial meningioma [10]. To date, however, the literature still contains no articles that describe scalp temperature changes in the symptomatic areas. To provide more information about nummular headache, we report herein that we have encountered three cases with scalp temperature changes in the symptomatic areas in our clinical practice. These observations might have implications regarding the pathogenesis of nummular headache.

## Case presentation

### Case 1

A 74-year-old female presented at our headache clinic with a 2-year history of headache that felt cold in the symptomatic area, which was confined to an ellipse 3 cm in diameter in the left parietal region. The headache consisted of stabbing pain of mild intensity. The episodes of pain lasted approximately 3 to 5 min each and occurred 2 to 3 times every week, with the intensity fluctuating around 2 to 3 on a 10-point visual analogue scale (VAS). There were no other complaints and no related focal neurological symptoms. The patient had no known family history of migraine, stroke, psychiatric disorders or dementia. She had a 5-year history of Type 2 diabetes. She had no cutaneous abnormalities in the painful area and had normal routine blood analyses, erythrocyte sedimentation rate and cerebral computed tomography. During the course of diagnosis and treatment, the patient’s headaches had occurred 4 times in the same area, and she complained of a cold sensation in the symptomatic skin. The symptomatic area was colder than the normal area, as estimated by touch and measured by an infrared thermometer. The recorded temperatures are presented in Table 1. As of a two-week follow-up visit, the patient had achieved good relief with gabapentin.

**Table 1:** Scalp temperature between symptomatic area and normal area

	Case1		Case 2		Case 3	
	Sym	Nor	Sym	Nor	Sym	Nor
1	35.8	36.4	37.3	36.8	37.4	36.8
2	36.1	36.5	37.4	36.7	37.3	36.7
3	35.5	36.5	37.4	36.8	37.4	36.8
4	35.5	36.5	37.3	36.6	37.5	36.8

*P*<0.01,the difference is statistically significant

Note: unit: °C。 Sym refers symptomatic area. Nor refers normal area

All measurements were made by reseacher

### Case 2

A 46-year-old male driver visited our outpatient headache clinic and complained of a twenty-year history of focal episodic pain located in a circumscribed area on the right temple. We learned that this patient had no personal or family history of migraine, stroke, hypertension or psychiatric disorders. The patient’s neurological examination was normal, with neither tenderness nor trophic changes inside the painful area. Blood tests and an MRI scan of the brain were also normal. The patient complained of an occasionally annoying hot sensation that appeared in the symptomatic area every time the pain attacked. Other than minor benefits from acupuncture and gabapentin, the intensity of the pain did not change with time. During the man’s visit to our clinic, we tested his scalp temperature using an infrared thermometer and recorded the results. We followed up with the patient over the next month and recorded the temperature the of the symptomatic area; here, we have opted to report only the records from the four most recent time points (Table 1).

### Case 3

A 38-year-old female patient complained of a two-month history of headache; the painful area was perfectly circular and confined to a diameter of approximately 3 cm on the posterior occipital part of the head. The pain was pulsating, sometimes stabbing, with the intensity fluctuating around 5 on a 10-point VAS. The attacks of pain differed in duration, sometimes lasting 5 min and sometimes lasting a whole day; fortunately, the intensity of the pain did not affect the patient’s life quality and did not cause a mood disorder. In addition, the patient complained that she had a hot sensation in the symptomatic area every time the pain attacked, particularly in the summer, and she could feel an obvious distinction between the symptomatic area and normal areas by touch. We tested the symptomatic area with an infrared thermometer and recorded the temperature every time when the patient visited our outpatient headache office. We reported the last four data points in Table 3. However, she had no other accompanying symptoms and no related focal neurological symptoms. She had a few years’ history of alcohol intake. She had no related family history of stroke, migraine, heart disease or psychiatric disorders. In the two months preceding her visit, the patient was given acupuncture and ibuprofen, which elicited a good response, but the frequency of the attacks did not change. The patient needed to take medicine or undergo acupuncture treatment whenever the pain attacked. The patient’s neurological examination was normal, with neither tenderness nor trophic changes in the painful area. Blood tests and an MRI scan of the brain were also normal.

## Discussion and conclusions

Here, we are the first to report three cases of scalp temperature changes in symptomatic areas affected by nummular headache, which may provide an enriched understanding of the variation in clinical manifestations of nummular headache. Scalp temperature changes are a form of paraesthesia, which may have clinical implications for its peripheral pathogenesis. The exact pathogenesis of nummular headache has not been clearly defined. Studies have advanced two main hypotheses: a peripheral mechanism and a central mechanism. Pareja and his colleagues have classified nummular headache as a localized neuralgia stemming from epicranial tissues – specifically, neuralgia affecting a terminal branch of one of the cutaneous nerves of the scalp [11]. The skin and hair changes in the symptomatic area also reflect a peripheral nerve lesion or, alternatively, an autoimmune process [12, 13]. In an extensive case series, Cuadrado and his colleagues reported that nummular headache was associated with a local increase in pain sensitivity to mechanical stimulation (reduced pressure pain thresholds [PPTs]) confined to the symptomatic area [14]. Although the pain is asserted to originate from epicranial tissues including the skull, scalp, vessels, and nerves [15–17], some nummular headaches have been described as “symptomatic nummular headaches”, which implies that they might be secondary to underlying structural lesions or other causes, such as subtentorial meningioma [10], pituitary lesion [18], or arachnoid cysts [19]. These observations represent the secondary form of nummular headache. Some researchers have also reported that nummular headache may arise from intracranial lesions or stress, both of which need to be ruled out when this type of headache appears [19]. Aside from these pathogenetic hypotheses, some researchers have suggested that nummular headache is caused by psychiatric disturbances [15]. However, other researchers have excluded the possibility of psychological factors as a cause of nummular headache disorder, and it is important to note that depression and anxiety are not symptoms of nummular headache disorder. Patients suffering from nummular headache disorder showed mood states similar to those of a healthier person [20]. We have discussed the pathogenesis of nummular headache, and we believe the observed changes in scalp temperature might be a clue in this mystery. However, we could not absolutely exclude the central hypothesis or the possibility of a complex pathogenesis involving both peripheral and central mechanisms. Further studies are still needed to help unveil the mystery.

In the initial report by Pareja in 2002, nummular headache was established to be a primary disease with clear-cut clinical features including a single, circumscribed location and mild to moderate pain. The three cases that we report in this paper involved paraesthesia, with the affected areas of the scalp being colder or warmer than the normal areas. This finding might support a peripheral mechanism of nummular headache. However, we could not definitively exclude the central hypothesis [21], and future studies with larger samples and follow-up designs may reveal a central mechanism as the cause of nummular headache. The ultimate judgement must be made by the appropriate healthcare professional(s) responsible for decisions regarding specific clinical procedures and treatment plans.

## Abbreviations

MRI: Magnetic Resonance Imaging
PPTs: Pressure Pain Thresholds
VAS: Visual Analogue Scale
